# Unidirectional All-Cellulose Composites from Flax via Controlled Impregnation with Ionic Liquid

**DOI:** 10.3390/polym12051010

**Published:** 2020-04-28

**Authors:** Feng Chen, Daisuke Sawada, Michael Hummel, Herbert Sixta, Tatiana Budtova

**Affiliations:** 1 Department of Bioproducts and Biosystems, School of Chemical Engineering, Aalto University, 00076 Aalto, Finland; feng.chen@aalto.fi (F.C.); daisuke.sawada@aalto.fi (D.S.); michael.hummel@aalto.fi (M.H.); herbert.sixta@aalto.fi (H.S.); 2 MINES ParisTech, PSL Research University, Center for Materials Forming-CEMEF, UMR CNRS 7635, CS 10207, 06904 Sophia Antipolis, France

**Keywords:** natural fibers, ionic liquid, composites, mechanical properties, structure–property correlations

## Abstract

Mechanically strong all-cellulose composites are very attractive in the terms of fully bio-based and bio-degradable materials. Unidirectional flax-based all-cellulose composites are prepared via facile room-temperature impregnation with an ionic liquid, 1-ethyl-3-methyl imidazolium acetate. To determine the optimal processing conditions, the kinetics of flax dissolution in this solvent is first studied using optical microscopy. Composite morphology, crystallinity, density, the volume fraction of cellulose II and tensile properties are investigated, indicating that flax dissolution should be within certain limits. On the one hand, the amount of cellulose II formed through dissolution and coagulation should be high enough to “fuse” flax fibers, resulting in a density increase. On the other hand, only the surface layer of the fibers should be dissolved to maintain the strength provided by the inner secondary layer and avoid a detrimental decrease in crystallinity. The highest Young’s modulus and strength, 10.1 GPa and 151.3 MPa, respectively, are obtained with a crystallinity of 43% and 20 vol% of cellulose II.

## 1. Introduction

An increasing demand for new eco-friendly materials has led to a significant development in the field of bio-based and bio-degradable composites as sustainable alternatives to petroleum-based materials. Natural fibers are renewable and are widely used as fillers and reinforcing matter in polymer composites. However, in most cases, the composite matrix is still based on oil-derived polymers. A special class of composites is all-cellulose composites (ACCs), which are composed of a cellulose matrix reinforced with cellulose fibers [1]. Based on the principle of all-polymer composites, ACCs overcome the problem of a weak fiber/matrix adhesion when fibers and matrix are made of different substances. Moreover, all-cellulose composites are 100% bio-based and bio-degradable [2,3,4].

Traditional polymer and all-polymer composites are usually produced by melt processing. Cellulose, as non-meltable polymer due to its extensive intramolecular and intermolecular hydrogen bonds, requires alternative processing techniques [3,5]. ACCs are generally manufactured either by the partial dissolution of the cellulose fibers’ surface to “splice” the fibers together upon coagulation (one-step approach) or by dispersing the cellulose fibers in a cellulose solution (two-step approach) [1,6,7,8]. In the first approach, the major continuous phase is cellulose fibers, whereas in the second approach, the composite body consists of a cellulose II matrix. In both cases, the cellulose solvent is removed by washing (typically in water) before the sample is dried.

To date, most efforts concerning the production of ACCs have followed the first approach. These ACCs were made using different cellulose solvents, dissolution times, temperatures and pressures (during drying) [9,10,11,12]. The influence of the dissolution time on the structure and properties of ACCs is not well established yet. For example, the aligned ramie fibers were immersed in lithium chloride/N, N-dimethylacetamide (LiCl/DMAc) for 1 to 12 h; exceptional longitudinal tensile properties (460 MPa tensile strength and 28 GPa Young’s modulus) were obtained after 2 h immersion [12]. Ramie is one of the strongest natural fibers with a 500−1000 MPa tensile strength and 20−60 GPa Young’s modulus for a single fiber [13,14]. When a conventional filter paper was impregnated with the same solvent, much more time, 12 h, was needed to get the strongest (within that study) all-cellulose composites with a tensile strength of 211 MPa and Young’s modulus of 9 GPa. However, when a filter paper was impregnated with 1-butyl-3-methylimidazolium chloride, 2.5 h were needed to reach the maximum values of around 90 MPa and 5 GPa for the tensile strength and Young’s modulus, respectively [15]. It was also demonstrated that the structural integrity of the cell wall of natural fiber can be severely affected by partial dissolution, which is detrimental for the mechanical properties of natural fiber-based ACCs [16]. It is known that the dissolution of natural fibers is influenced by fiber type, morphology and composition, as well as solvent type and temperature [17]. Therefore, the understanding of the dissolution kinetics of a given fiber in a certain cellulose solvent and correlation with fiber morphology are important to select optimal processing conditions for making ACCs with the best possible mechanical properties.

In terms of cellulose solvent options, LiCl/DMAc, NaOH-water and imidazolium-based ionic liquids (ILs) are the solvents most commonly used to prepare ACCs (see, for example, [4,12,18]). In particular, ILs attracted attention as cellulose solvents due to their high dissolution power, low vapor pressure and high thermal stability [19]. One of the advantages of ILs is that some are capable of dissolving cellulose even at room temperature, which is the case for 1-ethyl-3-methylimidazolium acetate ([EMIM][OAc]). However, some drawbacks of imidazolium-based ionic liquids should be taken into account if they are to be used for cellulose processing: for example, in the presence of impurities in the solvent or lignin in the pulp, side reactions may occur at temperatures around 100 °C [20,21]. Table 1 lists ACCs made from different sources of cellulose and ILs. The majority of studies involve heating the IL, even [EMIM][OAc], when manufacturing ACCs, most probably supposing that the decrease in solvent viscosity should reduce the dissolution time (see Table 1).

As a technical natural fiber and one of the strongest plant fibers, flax has been considered as a promising alternative to classical reinforcing glass fibers. The mechanical properties of flax fiber reported in the literature are 600–2000 MPa in terms of tensile strength, around 3% in terms of fracture strain, and 12–100 GPa in terms of the Young’s modulus [29,30]. To date, only isotropic textile- and non-woven mat flax-based ACCs have been studied [16,25]. Roughly, the reported properties of these ACCs are up to 80 MPa and 5 GPa in terms of strength and the Young’s modulus, respectively, which are only around one tenth of the corresponding values of single flax fibers.

In this work, room-temperature IL [EMIM][OAc] was used as solvent to prepare flax-based unidirectional ACCs by a selective dissolution method. The goal was to optimize the processing conditions to obtain ACCs with the best possible mechanical properties. We hypothesize that the too “profound” dissolution of a flax fiber, involving the destruction of the fiber’s secondary wall, can be detrimental for the mechanical properties of ACCs. To avoid this, we used optical microscopy to first study the kinetics of flax fiber dissolution in [EMIM][OAc] at different temperatures. Then, fibers were aligned and impregnated with [EMIM][OAc] under various conditions. Composite morphology and crystallinity were correlated with tensile mechanical properties and compared with those of other flax-based composites known from the literature.

## 2. Materials and Methods

### 2.1. Materials

Flax roving yarns with low twist degrees were obtained from the plant stem. A commercially available room temperature ionic liquid [EMIM][OAc] (purity > 95%) was purchased from IoLiTec. The moisture content was 0.27%, as determined by Karl Fischer titration. All materials were used as received.

### 2.2. Methods

#### 2.2.1. Chemical Composition of Fibers

The composition (carbohydrates and total lignin) of flax fibers was determined according to the NREL/TP-510-42618 norm. The amount of carbohydrates was detected by high performance anion exchange chromatography with pulse amperometric detection (HPAEC-PAD) using a Dionex ICS-300 system. Cellulose and hemicellulose contents were calculated according to the amount of monosaccharides following the Janson formula [31]. The acid-soluble lignin was determined using a Shimadzu UV 2550 spectrophotometer at a wavelength of 205 nm using an absorption coefficient of 110 L.g^−1^.cm^−1^. Additionally, the moisture content in as-received flax was determined using vacuum oven drying until a constant weight was achieved at 60 °C for 72 h.

#### 2.2.2. Optical Microscope Observation of Fiber Evolution in Ionic Liquid

One elementary flax fiber was placed between two glass plates (the distance in between was around 140 µm) with the ends fixed by tape and solvent added. The distance between the two glass slides, fixed by spacers, was much larger than the fiber’s thickness. The evolution of the fiber’s diameter was recorded by a DM4500P (Leica) optical microscope, in transmission mode, equipped with a Linkam TMS 91 hot stage to control temperature and a CCD camera (Metallux 3, Leitz, Wetzlar, Germany). Images were taken at various times. The relative diameter was calculated as D_t_/D_0_, where D_0_ and D_t_ are the initial diameter of the fiber at time 0 and diameter of the fiber at time t, respectively.

#### 2.2.3. ACC Preparation

**Figure 1:**
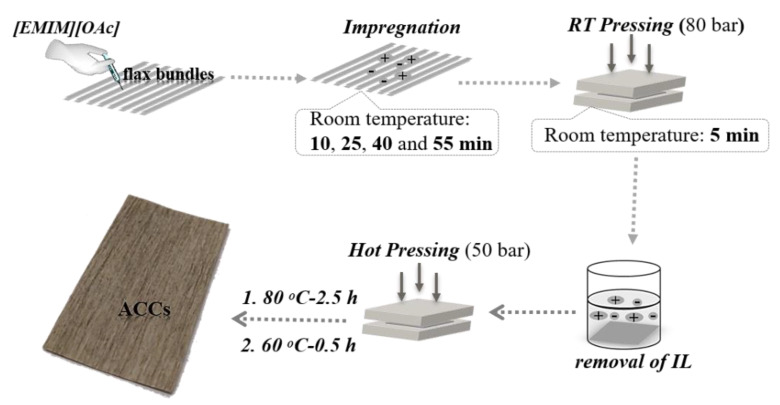
Manufacturing scheme for ACCs and examples of ACCs obtained.

Flax yarns were unidirectionally aligned in two layers (around 145 yarns, 3 g in total) on a Teflon mold and fixed at both ends to prevent shrinkage and distortion. The ACC manufacturing method is illustrated in Figure 1. Fibers were impregnated with 20 mL of [EMIM][OAc], kept under ambient conditions for desired times (10, 25, 40 and 55 min) and then pressed for 5 min (80 bar, room temperature) to complete the impregnation. The total duration of fiber treatment with ionic liquid was thus 15, 30, 45 and 60 min. Then, the sample was washed in a large amount of deionized water for 72 h to remove the ionic liquid from the system. Subsequently, the specimen was dried by hot pressing (50 bar, at 80 °C for 2.5 h and then at 60 °C for 0.5 h). A temperature gradient during drying was used to prevent the warping and/or heterogeneous contraction of the sample. In particular, decreasing the temperature from 80 °C to 60 °C rather than directly taking out the sample from the press at 80 °C allowed a gradual release in the inner stresses during drying.

#### 2.2.4. Calculation of Composite Apparent Density

The apparent or bulk density of obtained ACCs was calculated from the measurements of the sample volume (dimensions measured using L&W Micrometer, Lorentzen & Wettre Products, ABB, Kista, Sweden) and weight. For each sample, five measurements were performed and the average values were calculated.

#### 2.2.5. Scanning Electron Microscopy (SEM)

The morphology of the initial flax and of the ACCs was observed using a scanning electron microscope (Zeiss Sigma VP FE-SEM, Jena, Germany) at an accelerating voltage of 4 kV. Prior to examination, the surface and cross section of the specimen were coated with a thin layer of gold.

#### 2.2.6. X-ray Diffraction (XRD)

XRD patterns of flax fiber and ACCs were collected on an X-ray diffractometer (a X’Pert Pro MRD, PAN’alytical, Lelyweg, The Netherlands) with Cu-Kα radiation (λ  =  1.5418 Å) in reflection mode. Flax fiber and ACC were ground to powders and the samples were mounted on a multi-position sample holder. Data were collected in a 2θ range between 5° and 50°.

The amorphous contribution was evaluated using a smoothing method [32,33], applying the Savitzky–Golay [34] filter in the 2θ range from 8° to 45° for each diffraction profile. The window size and polynomial order for the Savitzky–Golay filter were set to 51 (corresponding to 4° by 2θ) and 1, respectively. The iteration for the background estimation was repeated until the iteration did not reduce the background area significantly. In this experimental and smoothing condition, the iteration was repeated 50 times. The crystallinity index (CRI) of flax fiber and of ACCs was estimated using the ratio of the area of total intensity (S_total_) to that of background intensity (S_bkg_) in the 2θ range from 10° to 32°:

(1) CRI=100×(1−SbkgStotal)

The background-corrected profile was fitted with pseudo-Voigt functions for (i) 1–10 (II), 1–10 (I) and 110 (I); (ii) 102/012 (I) and 110 (II); and (iii) 200 (I) and 020 (II), where the parenthesized numbers denote the crystalline phases of cellulose. The latter two peaks were not deconvoluted into the individual peaks because the fitting only aims to obtain intensities of 1–10 peaks for cellulose I and cellulose II. The software LMFIT [35] was used for the fitting. As the mass absorption coefficients are identical for crystalline polymorphs, quantitative phase analysis was performed using the single peaks of cellulose polymorphs [36]. The intensities for the 1–10 lattice plane of cellulose I (*I*_1–10(1)_) and cellulose II (*I*_1–10(2)_) can be expressed as a function of the structure factor, the volume of unit cell, and the volume fraction of each phase in the total cellulose crystal, as follows:

(2) $$I_{1-10\left(\alpha \right)}=\frac{K}{V_{\left(\alpha \right)}{}^{2}}{\left|F_{1-10\left(\alpha \right)}\right|}^{2}v_{\alpha }$$

where K is a constant for the instrument and sample, α is either cellulose I or II crystal, *V_(α)_* is unit cell volume, *F_1–10(α)_* is the structure factor and v_α_ is the volume fraction of crystalline phase α.

Thus,

(3) $$\frac{v_{2}}{v_{1}}=\frac{2.27\;I_{1-10\left(2\right)}}{I_{1-10\left(1\right)}}$$

where the coefficient 2.27 was calculated from the unit cell volume and the structure factor for the 1–10 lattice of cellulose I [37] and cellulose II [38]. This equation was used to calculate the volume fraction of cellulose II over total crystalline cellulose as follows:

(4) $$Cell_{II}\;vol\;\%=\frac{v_{2}}{v_{1}+v_{2}\;}$$

#### 2.2.7. Tensile Testing

The determination of the linear density (titer) and tenacity of a single flax fibers from as-received flax yarn were performed by using a Vibroskop 400 and Vibrodyn 400 (Lenzing Instruments GmbH & Co KG, Austria) at 23 °C and 50% relative humidity (RH) The settings for the Vibrodyn 400 were gauge length: 20 mm and strain rate: 10 mm/min. Ten elementary fibers were measured to gain the mean values of titer, tenacity and elongation. The elastic modulus of the fibers was calculated from the slope of the entire elastic region of the stress–strain curves by using a MATLAB script according to ASTM standard D2256/D2256M.

The mechanical properties of flax-based ACCs were studied using an Instron 4204 Universal Tensile Tester (INSTRON, Buckinghamshire, UK). At least five specimens (50 × 5 mm, length and width, respectively) with known thickness of each formulation were analyzed. Samples were conditioned for 24 h in a controlled environment of 50% RH and 23 °C. The measurement was carried out at a gauge length of 20 mm and an extension rate of 10 mm/min with a load of 1 kN.

## 3. Results and Discussion

### 3.1. Analysis of Flax Fibers

**Figure 2:**
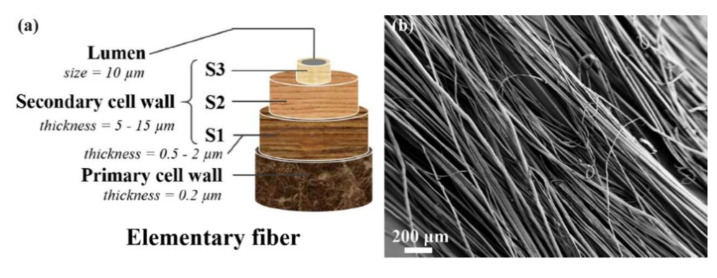
(**a**) Schematic representation of the multi-scale composite structure of flax elementary fiber (adapted from [40]) and (**b**) SEM image of the as-received flax fibers.

It is known that the cell wall of an elementary flax fiber is composed of hierarchically organized layers (see Figure 2a). Two outer layers (primary and S1 layers) contain less ordered and poorly oriented cellulose. To the contrary, the highly ordered and densely packed inner S2 layer plays an important role in the mechanical performance of flax fibers [39]. The scanning electron micrograph of the as-received flax fibers is shown in Figure 2b; the average diameter of an elementary flax fiber is around 20 µm. The mechanical properties and composition of the flax used in this work are summarized in Table 2; a representative stress–strain curve of elementary flax fibers can be seen in Figure S1 of the Supporting Information. The results obtained are in line with what was already reported for flax [29,30].

When converting natural fibers into ACCs, the strategy is to partially dissolve the surface layer to transform it into the matrix phase while maintaining the fiber “core”, in particular, the S2 layer, to provide good mechanical properties. Therefore, optical microscopy studies were performed to follow the evolution of flax fiber dissolution over time in [EMIM][OAc] in order to select the optimal conditions for making ACCs.

### 3.2. Fiber Size Evolution in [EMIM][OAc]

**Figure 3:**
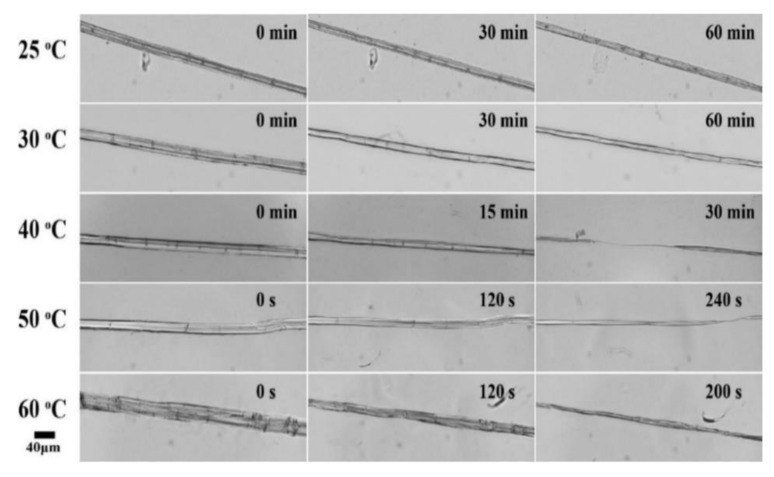
Optical microscope images of a flax fiber radially dissolving in ionic liquid (IL) at various temperatures.

**Figure 4:**
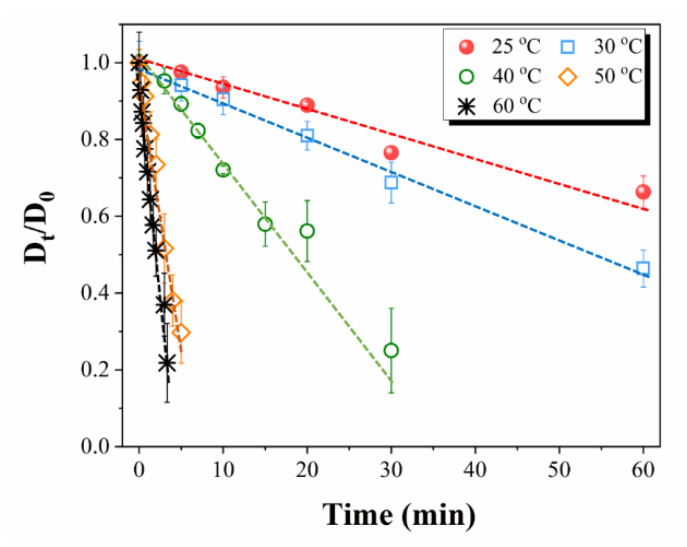
Relative diameter of flax fiber in [EMIM][OAc] as a function of time at various temperatures. Dashed lines are linear approximations.

An example of the evolution of an elementary flax fiber in [EMIM][OAc] observed by an optical microscope is depicted in Figure 3. Flax is radially dissolving without swelling or ballooning, even at room temperature, indicating that [EMIM][OAc] is a good solvent for flax, similar to NMMO monohydrate [41]. The relative diameter of a flax fiber in IL as a function of time at various temperatures is presented in Figure 4. Temperature increase strongly accelerated the dissolution of flax: for example, within 30 min, the diameter was reduced by 20% at 25 °C, and it was reduced by almost 80% at 40 °C. At higher temperatures, above 50 °C, the fiber was dissolved within 10 min.

The dissolution rate of flax in [EMIM][OAc] was calculated as a slope to each set of experimental data approximated with a linear dependence (Figure 4). We tested if the dissolution rate can be approximated by an Arrhenius type law, similar to the rates of reactions:

(5) $$dissolution\;rate\;\sim \exp\left(\frac{E}{RT}\right)$$

where *E* is the activation energy, R is the universal gas constant and *T* is temperature in K. Figure S2 in the Supporting Information shows that Equation (5) describes well the dissolution rate calculated from experimental data. The dissolution rate of fibers should be taken into account when making all-cellulose composites. For example, it has been shown that the immersion of ramie fibers in DMAc/LiCl for a long time resulted in a dramatic decrease in ACC mechanical properties [12].

The results for the kinetics of flax dissolution suggest using room temperature for the preparation of ACC using [EMIM][OAc]. At higher temperatures, the dissolution will be too quick, resulting in a high fraction of mechanically “weaker” amorphous cellulose and cellulose II (with a theoretical tensile strength of around 800–1000 MPa [42,43], as compared to much “stronger” cellulose I with a theoretical tensile strength of 13–17 GPa [44]). Given the hierarchically layered structure of flax elementary fibers and the aim of producing high strength ACC, the dissolution of only primary and S1 layers is preferred, for keeping the mechanically rigid inner S2 layer untouched. The primary and S1 layers represent about 10% of the total thickness of the flax elementary fiber. According to Figure 4, they are dissolved within about 20 min at room temperature, this being applied to an elementary fiber alone. When impregnating many fibers closely aligned to prepare ACCs, slower dissolution is expected. Solvent diffusion decreases rapidly as more and more cellulose dissolves, and there is no convection-promoting mass transfer. The impregnation times selected for making all-cellulose composites at room temperature were thus 15, 30, 45 and 60 min.

### 3.3. Morphology of the ACCs

**Figure 5:**
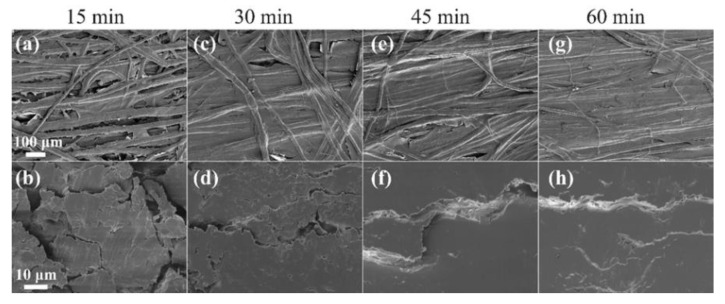
Scanning electron micrographs of the surface (**a**,**c**,**e** and **g**) and cross-section (**b**,**d**,**f** and **h**) of flax-based ACCs after 15, 30, 45 and 60 min of impregnation time at room temperature, respectively.

The morphologies of the ACCs impregnated for different times are presented in Figure 5. After 15 min of impregnation (Figure 5a,b), the single fibers are still visible; they are only slightly fused together. The composite surface and cross-section show voids between the fibers. A time of 15 min does not allow the dissolution of enough cellulose for it to act as an “adhesive” upon coagulation. As the impregnation time is increased, the fibers “fuse” gradually and their contours in the SEM images are visibly reduced. After 45 min, the cross-section appears mostly homogeneous, indicating that enough cellulose has been dissolved to fill the voids between the fibers and form a continuous matrix (Figure 5e,f).

### 3.4. Crystallinity of the ACCs

**Figure 6:**
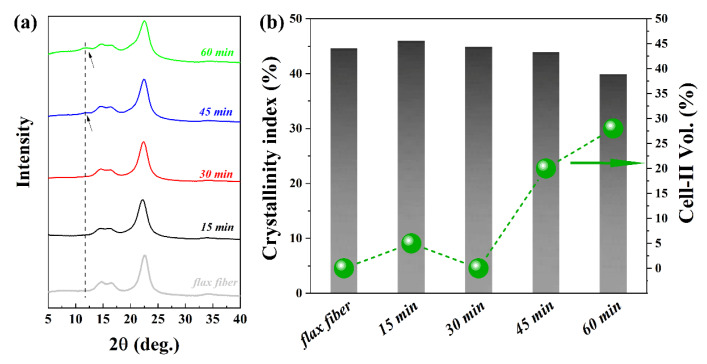
(**a**) X-ray diffractograms of flax fibers and ACC (the arrow shows the appearance of cellulose II) and (**b**) total crystallinity and volume fraction of cellulose II in ACCs as a function of impregnation time.

The X-ray diffractograms of flax fibers and flax-based ACCs are shown in Figure 6a. The main diffraction peaks of flax are typical for cellulose I from higher plant cellulose [45]. The flax fibers contained cellulose I with a crystallinity of 44.6%. These peaks remain in all of the ACC XRD profiles, indicating the presence of cellulose I (Figure 6a). After short impregnation times, no apparent difference in the XRD profiles is seen between flax fibers and ACCs made with 15 and 30 min of impregnation (Figure 6a). The crystallinity remains nearly constant at about 45%, and the volume fraction of cellulose II (Figure 6b) is still around zero (within the accuracy of the method). This is because only pectin, hemicellulose and low molecular weight and amorphous cellulose in the outer layers, primary wall and S1 are initially dissolved and converted into matrix, causing a reduction in the diameter of the elementary fiber [46,47]. However, ACCs produced by 45 and 60 min of impregnation clearly show a cellulose II polymorph as revealed by the additional peak at around 2θ = 12.2° (Figure 6a). The volume fraction of cellulose II in relation to the total crystalline cellulose is 20% and 28% for 45- and 60-min impregnation, respectively (Figure 6b). Impregnation for 60 min decreased the crystallinity, indicating the dissolution of cellulose I crystalline domains in the S2 layer (Figure 6b).

### 3.5. Mechanical Performance of the ACCs

**Figure 7:**
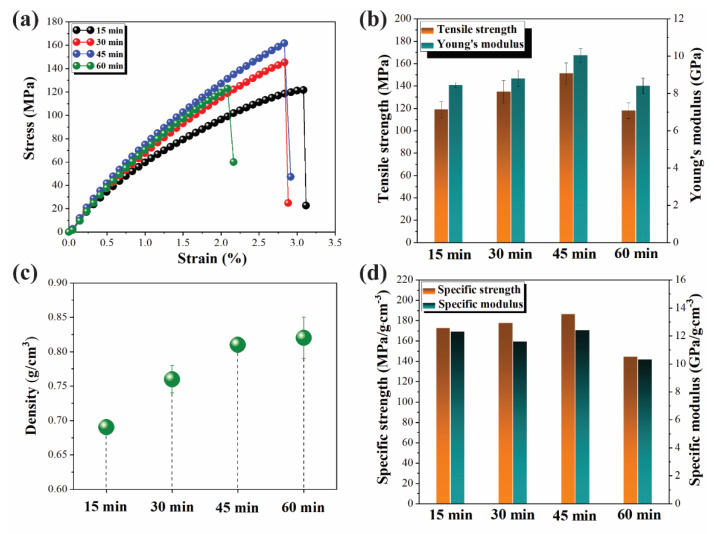
(**a**) Representative stress–strain curves for ACCs made with different impregnation times; (**b**) tensile strength and Young’s modulus of flax-based ACCs as a function of impregnation time; (**c**) density values of ACCs as a function of impregnation time; and (**d**) specific strength and specific modulus of flax-based ACCs as a function of impregnation time.

The mechanical performance of all ACCs was evaluated by tensile tests; the representative stress–strain curves in the longitudinal direction are shown in Figure 7a. The mean values of the tensile strength and Young’s modulus as a function of impregnation time are presented in Figure 7b; all values are given in Table S1 of the Supporting Information. Both tensile strength and Young’s modulus increase with increasing impregnation time and reach a maximum at 45 min with a tensile strength of 151.3 MPa and a Young’s modulus of 10.1 GPa. Thereafter, the mechanical properties drop. Density also increases with impregnation time and reaches a plateau at 45 min (Figure 7c). The density increase is due to the dissolved cellulose “filling” the voids between fibers, as shown in the SEM images (Figure 5). The specific mechanical properties (specific strength and specific modulus) were calculated and are shown in Figure 7d; both do not vary, being within the experimental errors, for 15–45 min impregnation, but decrease at 60 min.

The combination of the results obtained from tensile testing with the results of the X-ray analysis and SEM imaging shows that tensile properties of the ACCs depend on two counteracting effects. On the one hand, sufficient amounts of cellulose need to be dissolved and converted into a continuous matrix to act as cellulosic “adhesive” and “fill” the voids. The increase in the mechanical properties is governed by the increase in composite density. On the other hand, the overall crystallinity has to be preserved, and only minimum amounts of crystalline cellulose I domains should be converted to cellulose II or amorphous cellulose. The decrease in crystallinity with the increased volume of cellulose II is the reason for the decrease in the mechanical properties at 60 min of impregnation, as the densities after 45- and 60-min impregnation times are the same. Thus, optimum mechanical properties are reached through intermediate impregnation times that promote the dissolution of cellulose in the outer layer of the flax fiber but not in the secondary cell wall, to maintain the integrity of the cellulose I domains. The mechanical properties in the transverse direction are much weaker, as expected (see an example in Figure S3). A 30–40 fold drop in tensile strength in the transverse direction was also reported for ACCs based on ramie fibers [1]. It should be noted that small amounts of ionic liquid remained in the composites, around and below 1 wt. %, as determined by the presence of nitrogen in ACC by elemental analysis. The quantity of nitrogen in the initial flax was negligible, at around 0.06 wt. %.

To evaluate the obtained unidirectional flax-based ACCs, their mechanical properties were compared to isotropic flax-based ACCs [16,25] and unidirectional flax-reinforced polymer-based composites [48,49,50,51,52]. As shown in Table 3, the tensile strength of the obtained ACC not only exceeds that of isotropic flax ACC, as expected, but also of other unidirectional flax reinforced starch-, polylactic acid (PLA)-, epoxy- and polypropylene-based polymer composites.

**Figure 8:**
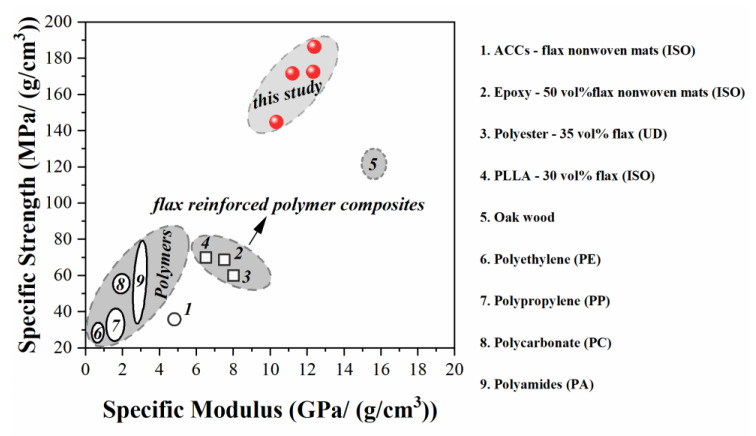
Comparison of the specific mechanical properties of unidirectional flax-based ACCs made in this work with those of other flax-based structures (1 [16]), flax-reinforced composites (2 [16], 3 [53] and 4 [53]), oak wood (5 [54]) and conventional polymers (6 [55], 7 [55], 8 [55] and 9 [55]). UD means unidirectional while ISO means isotropic.

The specific mechanical properties of the obtained flax-based ACCs are compared with those of other flax-based materials: flax reinforced polymer composites, oak wood and other polymers (Figure 8). The specific strength values range from 150 to 190 MPa/(g/cm^3^), with specific moduli of around 12 GPa/(g/cm^3^). The properties of the obtained flax-based ACC are superior to those of conventional polymer-based materials and composites and even oak wood. This highlights the potential of ACCs produced at room-temperature for “stiffness-driven” light-weight materials, where minimum structural weight is required.

## 4. Conclusions

All-cellulose composites based on unidirectional oriented flax fibers were prepared via impregnation with the room temperature ionic liquid [EMIM][OAc]. In order to select the optimal conditions for the preparation of an ACC with the highest mechanical properties, the kinetics of elementary fiber dissolution in this solvent were first studied at various temperatures. The results showed that at temperatures higher than 40 °C the dissolution is too quick, involving the destruction of the fiber secondary wall, the latter being the main component in flax providing the mechanical reinforcement. The performance of the ACC was demonstrated to depend on a compromise between the formation of sufficient matrix“gluing” fibers together and filling voids, resulting in a density increaseon the one hand, and the non-dissolution of the secondary wall and the restriction of the formation of lower crystallinity and mechanically weaker cellulose II on the other hand. The best values were obtained for the composite with 45 min impregnation, resulting in a mean value for maximal tensile strength of 151.3 MPa and a Young’s modulus of 10.1 GPa. The study revealed the importance of the understanding of the evolution of natural fibers in cellulose solvents, resulting in finding a suitable strategy for making high-performance ACCs and taking full advantage of the excellent mechanical properties of the cellulose fiber itself. The use of solvent at room temperature is not energy-consuming, making the process economically attractive. The values are better than those for many polymer composites reinforced with flax fibers, providing a promising way of making fully bio-based materials with excellent mechanical properties.

## Figures and Tables

**Table 1:** Summary of all-cellulose composites (ACCs) manufactured using the imidazolium-based ionic liquids 1-ethyl-3-methylimidazolium acetate [EMIM][OAc], 1-butyl-3-methylimidazolium chloride [BMIM]Cl and 1-butyl-3-methylimidazolium acetate [BMIM][OAc].

IL	Raw Materials	Impregnation Condition	Method	Ref.
[BMIM]Cl	Cotton fabric	100 °C (30 min) and 150 °C (hot press, 30 min)	One-step	[22]
	Hinoki lumber	100 °C (30 min) and 210 °C (hot press, 30 min)		
[BMIM]Cl	Jute fabric	110 °C (2–8 h)		[23]
	Filter paper			
[BMIM]Cl	Microfibrillated cellulose	80 °C (20, 40, 80 and 160 min)		[15]
[BMIM]Cl	Lyocell fabric	110 °C (3 h)		[9]
[BMIM]Cl	Lyocell fabric	110 °C (hot press, 0.5–4 h)		[11]
[BMIM][OAc]	Cordenka textile	95 °C (hot press, 60 min)		[24]
[BMIM][OAc]	Linen textile	110 °C (hot press, 80 min)		[25]
	Rayon textile			
[EMIM][OAc]	Paper	95 °C (10 s) and 95 °C (hot press, 0.5–6.5 h)		[26]
[EMIM][OAc]	Birch wood plies	95 °C (30 min)		[27]
[EMIM][OAc]	Silk/hemp/cotton thread	60 °C (5 min)		[28]
[BMIM]Cl	Lyocell nonwoven mats	103 °C (1 min)	Two-step	[16]
[EMIM][OAc]	Cordenka fabric	80 °C (hot press, 0.5–1 h)		[4]
	Flax nonwoven mats			

**Table 2:** Properties of the as-received elementary flax fibers.

Cellulose (%)	Hemicellulose (%)	Lignin (%)	Tensile Strength (MPa)	Young’s Modulus (GPa)	Strain (%)
83.3	11.3	2.3	1364 ± 283	59 ± 9	2.9 ± 0.5

**Table 3:** Comparison of the mechanical properties of unidirectional (UD) flax-reinforced polymer composites, isotropic (ISO) flax-based ACCs and the strongest unidirectional flax-based ACC obtained in this study.

Matrix	Flax Content	Longitudinal Tensile Strength (MPa)	Young’s Modulus (GPa)	Ref.
Starch	60 wt. %-UD	79	9.3	[48]
Polylactic acid	30 wt. %-UD	54	6.3	[49]
Epoxy	40 vol. %-UD	133	28	[50]
Epoxy	37 wt. %-UD	132	15	[51]
Polypropylene	30 wt. %-UD	13	6.1	[52]
Cellulose solution	50 wt. %-ISO	34	4.6	[16]
Flax-based ACC	100 wt. %-ISO	46	0.86	[25]
Flax-based ACC	100 wt. %-UD	151.3	10.1	This study

## Supplementary Materials

The following are available online at https://www.mdpi.com/2073-4360/12/5/1010/s1, Figure S1: Representative stress-strain curve of a single flax fiber, Figure S2: Arrhenius plot of dissolution rate as a function of inverse temperature, Figure S3: Representative stress-strain curve of flax-based ACCs after 45 min impregnation tested in transverse direction, Table S1: Properties of flax based ACCs.
