# Supplementary material for: Unidirectional All-Cellulose Composites from Flax via Controlled Impregnation with Ionic Liquid
Source: Polymers (Basel). 2020 Apr 28;12(5):1010. doi: 10.3390/polym12051010 (PMC7284595; doi:10.3390/polym12051010)
Supplement: Supplementary file 1 [file polymers-12-01010-s001.pdf]

polymers MDPI

Supporting Information

Unidirectional all-cellulose composites from flax via controlled impregnation with ionic liquid

Feng Chen[1], Daisuke Sawada[1], Michael Hummel[1], Herbert Sixta[1] and Tatiana Budtova[1,2]*

1 - Department of Bioproducts and Biosystems, School of Chemical Engineering, Aalto University, P.O. Box 16300, 00076 Aalto, Finland

2 - MINES ParisTech, PSL Research University, Center for Materials Forming-CEMEF, UMR CNRS 7635, CS 10207, 06904 Sophia Antipolis, France.

E-mail: Tatiana.Budtova@aalto.fi; Tatiana.Budtova@mines-paristech.fr

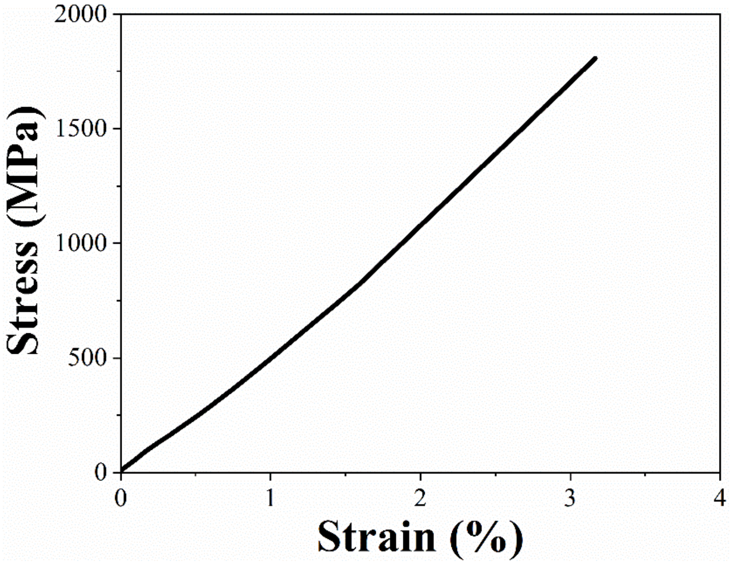

Figure S1. Representative stress-strain curve of a single flax fiber.

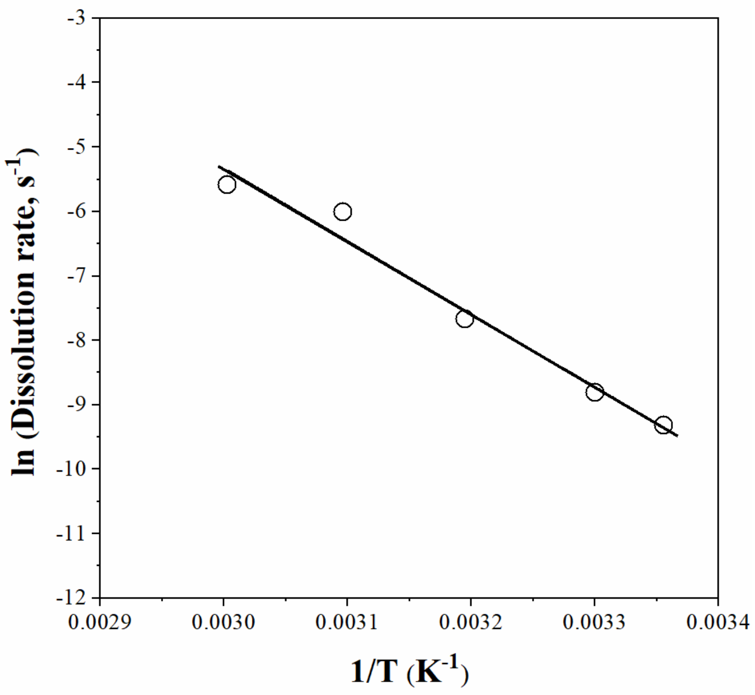

Figure S2. Arrhenius plot of dissolution rate as a function of inverse temperature.

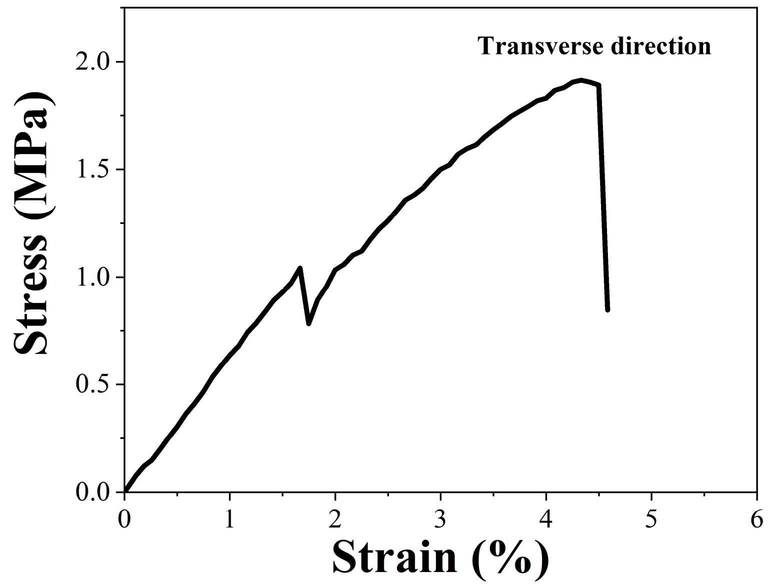

Figure S3. Representative stress-strain curve of flax-based ACCs after 45 min impregnation tested in transverse direction.

Table S1. Properties of flax based ACCs.

	15 min	30 min	45 min	60 min
Thickness (mm)	0.37 ± 0.03	0.30 ± 0.01	0.31 ± 0.02	0.30 ± 0.01
Density (g/cm^3)	0.69 ± 0.01	0.76 ± 0.02	0.81 ± 0.01	0.82 ± 0.03
Total crystallinity (%)	46	44.9	43.9	39.9
Volume fraction of Cell II (%)	0.05	0	20	28
Tensile Strength (MPa)	121.1 ± 7.9	134.9 ± 9.9	151.3 ± 9.5	117.9 ± 7
Young's modulus (GPa)	8.5 ± 0.1	8.8 ± 0.4	10.1 ± 0.4	8.4 ± 0.4
Strain at break (%)	3.0 ± 0.5	2.5 ± 0.3	2.7 ± 0.2	2.2 ± 0.1
Specific strength (MPa/g·cm^{-3})	172.6	177.5	186.4	144.4
Specific modulus (GPa/g·cm^{-3})	12.3	11.6	12.4	10.3
